# Early Initiation of Awake Veno-Venous Extracorporeal Membrane Oxygenation Can Attenuate Muscle Atrophy and Weakness in Acute Respiratory Distress Syndrome

**DOI:** 10.7759/cureus.9926

**Published:** 2020-08-21

**Authors:** Nobuto Nakanishi, Yuri Okamoto, Tetsuya Okahisa, Jun Oto

**Affiliations:** 1 Emergency and Critical Care Medicine, Tokushima University Hospital, Tokushima, JPN; 2 Respiratory Medicine and Rheumatology, Tokushima University Hospital, Tokushima, JPN; 3 Rehabilitation, Tokushima University Hospital, Tokushima, JPN

**Keywords:** ards (acute respiratory distress syndrome), vv ecmo, muscle atrophy, musculoskeletal rehabilitation, icu patients, titin, influenza virus type a, diaphragm dysfunction, early nutrition

## Abstract

Patients with acute respiratory distress syndrome (ARDS) exhibit prominent muscle atrophy and weakness. Although these patients often require deep sedation to perform lung-protective ventilation, extracorporeal membrane oxygenation (ECMO) can keep patients awake and make mobilization possible. A 60-year-old man was treated with ECMO due to ARDS. A multidisciplinary team conducted mobilization with standing on day 3. During intensive care unit (ICU) stay, catabolism was ongoing (urinary titin: 24.1-38.4 pmol/mg Cr), but the rectus femoris muscle, measured by ultrasound, moderately decreased by 5.3%, 10.8%, and 13.0% on days 3, 5, and 7, respectively, with maintained Medical Research Council score of 58-60. Diaphragm thickness remained unchanged. On day 5, he was separated from ECMO. After ambulation training, he was discharged from ICU on day 7. He returned home without prominent physical dysfunction. Our experience indicates early initiation of awake ECMO can accompany mobilization and attenuate muscle atrophy and weakness in ARDS.

## Introduction

The mortality of acute respiratory distress syndrome (ARDS) decreased from 35.4% to 28.3% in a decade [1]. Survivors of ARDS, however, suffer from prolonged physical dysfunction [2]. Regarding the six-minute walk test, the ability remains 49%, 64%, 66%, and 76%, at 3 months, 6 months, 12 months, and five years after discharge from the hospital [2]. Functional disability is caused by muscle atrophy acquired in the intensive care unit (ICU), which prolongs after discharge from the ICU [3]. Consequently, only 44% and 69% of patients can return to the job one year and five years later, respectively [4].

ARDS patients are treated with lung protective strategies including low tidal volume ventilation in order to prevent lung injury [5]. These strategies often require deep sedation and neuromuscular-blocking agents to provide better adaptation to the ventilator and allow a reduction of tidal volume. Although mobilization is important in patients with ARDS, sedation hampers the active mobilization of patients. Moreover, discontinuation of spontaneous breathing to prevent lung injury is deleterious to the diaphragm muscle [6]. Therefore, lung protective strategies may lead to prominent limb and diaphragm muscle atrophy, leading to prolonged physical dysfunction.

Extracorporeal membrane oxygenation (ECMO) can keep patients awake and make early mobilization possible in ARDS. Because respiratory ECMO can provide oxygen and remove carbon dioxide, the central respiratory drive can be attenuated without deep sedation and neuromuscular-blocking agents [7]. We experienced a case of a successfully rehabilitated patient in ARDS through the early initiation of ECMO without prolonged functional impairment. ECMO may be useful for preventing prominent muscle atrophy and weakness. The patient provided written consent to publish this case report.

## Case presentation

**Figure 1:**
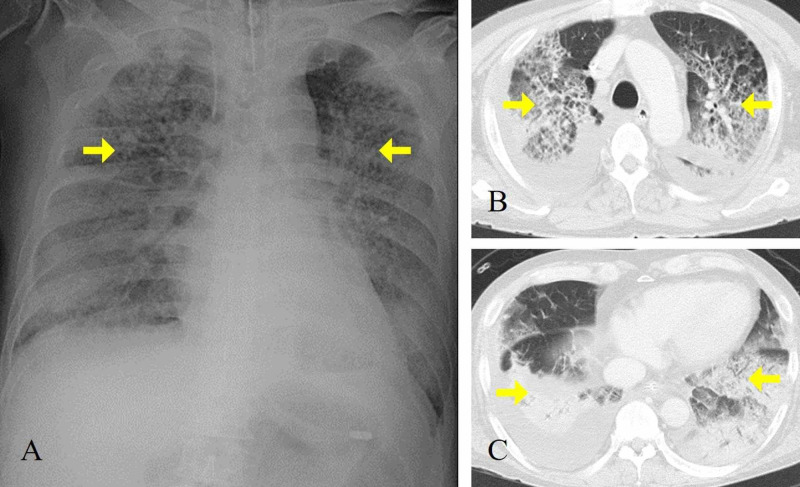
Chest X-ray and CT at ICU admission Chest X-ray (A) and CT (B, C) showing bilateral alveolar infiltrates with normal cardiac size consistent with acute respiratory distress syndrome.

A 60-year-old male (height: 173 cm; weight: 70.9 kg) with a past history of rheumatoid arthritis was transferred to the ICU due to ARDS caused by H1N1 influenza virus infection. In a previous hospital, he was treated for influenza and possible secondary bacterial pneumonia for 18 days. However, oxygenation rapidly deteriorated two days before the transfer with the suspicion of ARDS. After the ICU admission, he was immediately intubated and the mechanical ventilation was started in an A/C mode with a tidal volume of 6.7 mL/kg of predicted body weight, peak inspiratory pressure of 24 cmH_2_O, positive end-expiratory pressure (PEEP) of 14 cmH_2_O, fraction of inspired oxygen 1.0, respiratory rate (RR) of 21 breaths/min, during which blood gas values were as follows: pH 7.35, PaO_2_ of 103 mmHg, and PaCO_2_ of 46 mmHg. Chest X-ray and CT showed diffuse bilateral infiltration with normal left ventricular function on cardiac echocardiography (Figure 1).

Murray score was 3.25 (PaO_2_/FiO_2 _of 103, X-ray alveolar consolidation confined to 4 quadrants, PEEP of 14 cmH_2_O, compliance of 37 mL/cmH_2_O). In view of the deteriorating condition, we decided to start veno-venous ECMO using Cardiohelp (Maquet CP, Hirrlingen, Germany). Cannulation performed was with a 22-Fr cannula (PCKC-V, Toyobo, Japan) for drainage into his right atrium through the right femoral vein and with a 16.5-Fr cannula (CAPIOX EBS, Terumo, Japan) for blood infusion into the right internal jugular vein. The initial settings of ECMO begin at a flow of 42 mL/kg/min, speed of 3,000 rotations/min, fraction of delivered oxygen (FiO_2_) of 100%, and sweep of 2 L/min. The ventilator was changed to a lung-rest setting (PEEP of 10 cmH_2_O and pressure control of 8 cmH_2_O, RR of 15 breaths/min). In addition, he was empirically started on meropenem and azithromycin.

In the previous hospital, he was treated with methylprednisolone (1,000 mg/day) for two days. Additional methylprednisolone 1,000 mg was used for one day with subsequent steroid dosing (predonine 70 mg/day) in our hospital. The treatment of steroid pulse was performed because of suspected autoimmune disease or drug-induced pneumonia by methotrexate treatment. However, his blood test was finally negative for all autoimmune antibodies. Moreover, all bacteria and virus tests were negative. Therefore, the deteriorated patient’s oxygenation was considered to be suggestive of ARDS caused by influenza infection.

The multidisciplinary mobilization team with a physical therapist, nurse, medical engineer, and doctor evaluated the patient’s status and developed a daily plan of rehabilitation based on progressive mobilization protocol. On day 2, rehabilitation was limited to a passive range of motion due to the low conscious level (Richmond Agitation Sedation Scale [RASS] score: −2 to −4). Active mobilization was started from day 3 with patient consciousness alert (RASS score: −1 to 0). The patient was mobilized with sitting on the edge of bed and standing.

On day 4, mobilization level was elevated to standing two times in a day. On day 5, foot stepping was added to standing. On day 6 after the separation from ECMO, the patient was transferred from the bed to the chair with minimum assistance. On day 7 after extubation, the patient was ambulated in the ICU. Nutrition was started from day 2, and the intake protein increased to 1.3 g/kg on day 4 with calorie suppressed to 17.6 kcal/kg (Peptamen Intense®, Nestlé, Vevey, Switzerland). During mobilization in the ICU, the patient did not experience any hemodynamic, ventilatory, or cannulation trouble.

**Figure 2:**
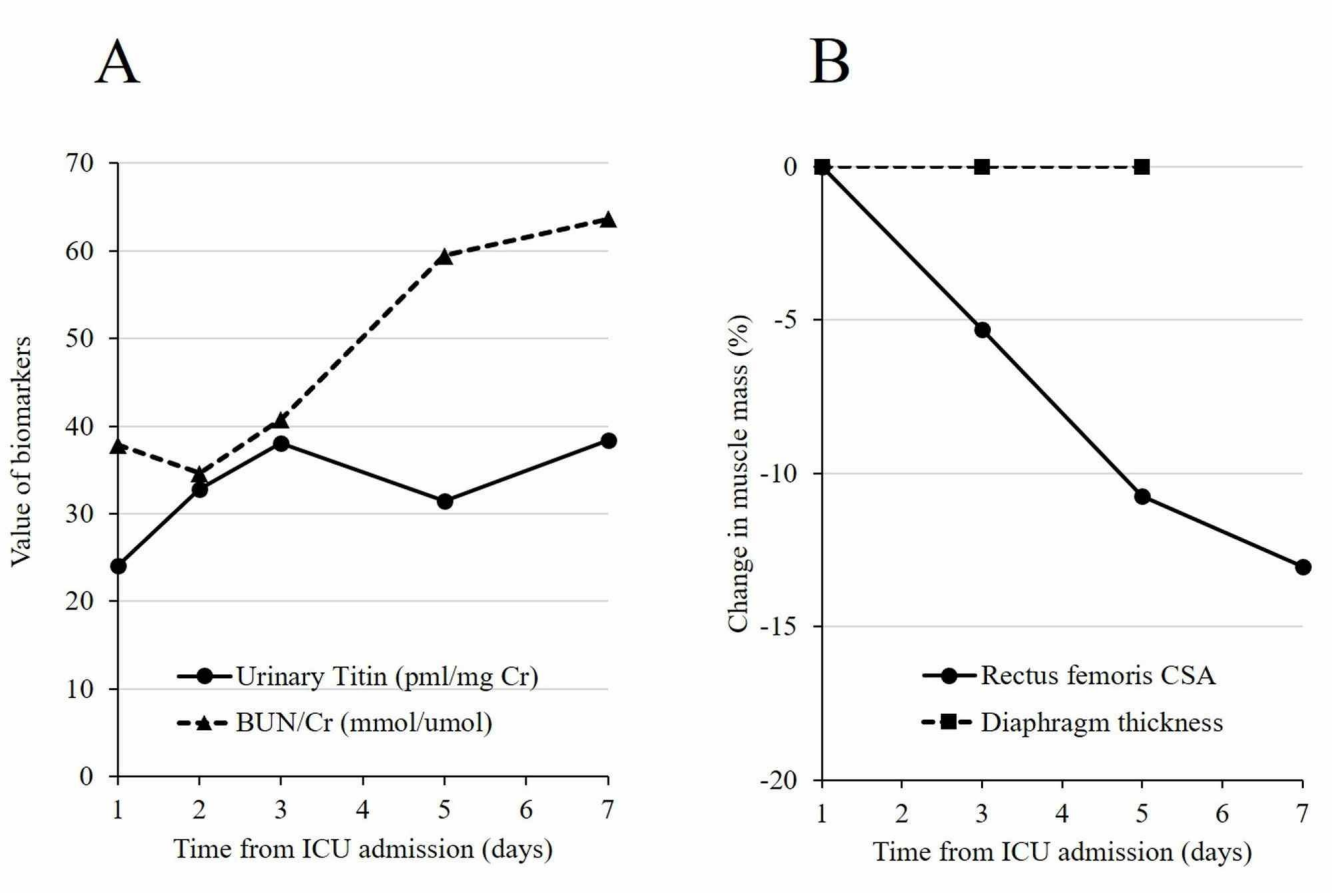
Catabolism and muscle atrophy Catabolism and muscle atrophy are shown after admission to the intensive care unit. (A) Urinary titin and BUN/Cr. (B) Rectus femoris cross-sectional area and diaphragm thickness. Abbreviations; ICU, intensive care unit; BUN, blood urea nitrogen; Cr, creatinine; CSA, cross-sectional area

By using ultrasound, muscle mass was measured three times, and the median value was recorded for evaluation. Experienced examiner (N.N.) conducted the measurement with previously reported intra- and inter-observer reproducibilities [8,9]. The Rectus femoris cross-sectional area was measured between the anterosuperior iliac spine and the proximal end of the patella [9]. Diaphragm thickness was also measured by the ultrasound from the zone of apposition [8]. During the seven days of admission, the rectus femoris muscle mass decreased by 5.3%, 10.8%, and 13.0% on days 3, 5, and 7, respectively, compared with the ICU admission value (Figure 2).

Diaphragm thickness remained unchanged (1.8 cm from day 1 through day 5) during five days of mechanical ventilation. The urinary titin, corrected with urinary creatinine, was measured using ELISA kit (27900 Titin N-Fragment Assay Kit, Immuno-Biological Laboratories, Gunma, Japan) to evaluate catabolism. Urinary titin level was much higher than the normal range (normal range: 1-3 pmol/mg Cr, urinary titin level in this patient: 24.1, 32.8, 38.0, 31.5, 38.4 pmol/mg Cr on days 1, 2, 3, 5, and 7, respectively), indicating ongoing catabolism. Blood urea nitrogen/creatinine (BUN/Cr) was also elevated to 34.6-63.6 (normal range: 10-20). In spite of the catabolism, the Medical Research Council (MRC) score was maintained at 59, 60, 58, and 60 on days 2, 3, 5, and 7, respectively. Furthermore, the patient did not experience ICU-acquired weakness [6].

During ECMO, the patient’s tidal volume was reduced to 4.0-4.6 mL/kg, with peak inspiratory pressure of 18 cmH_2_O and PEEP of 10 cmH_2_O. His lung condition gradually improved. The bilateral infiltration of the X-ray was clearly disappearing. On day 4, oxygenation had significantly improved to PaO_2_/FiO_2_ of 183. On day 5, he was completely separated from ECMO. On day 6, he was liberated from mechanical ventilation. On day 7, he was discharged from the ICU. The functional status score for the ICU (FSS-ICU) was 31 out of 35 at discharge from the ICU.

**Figure 3:**
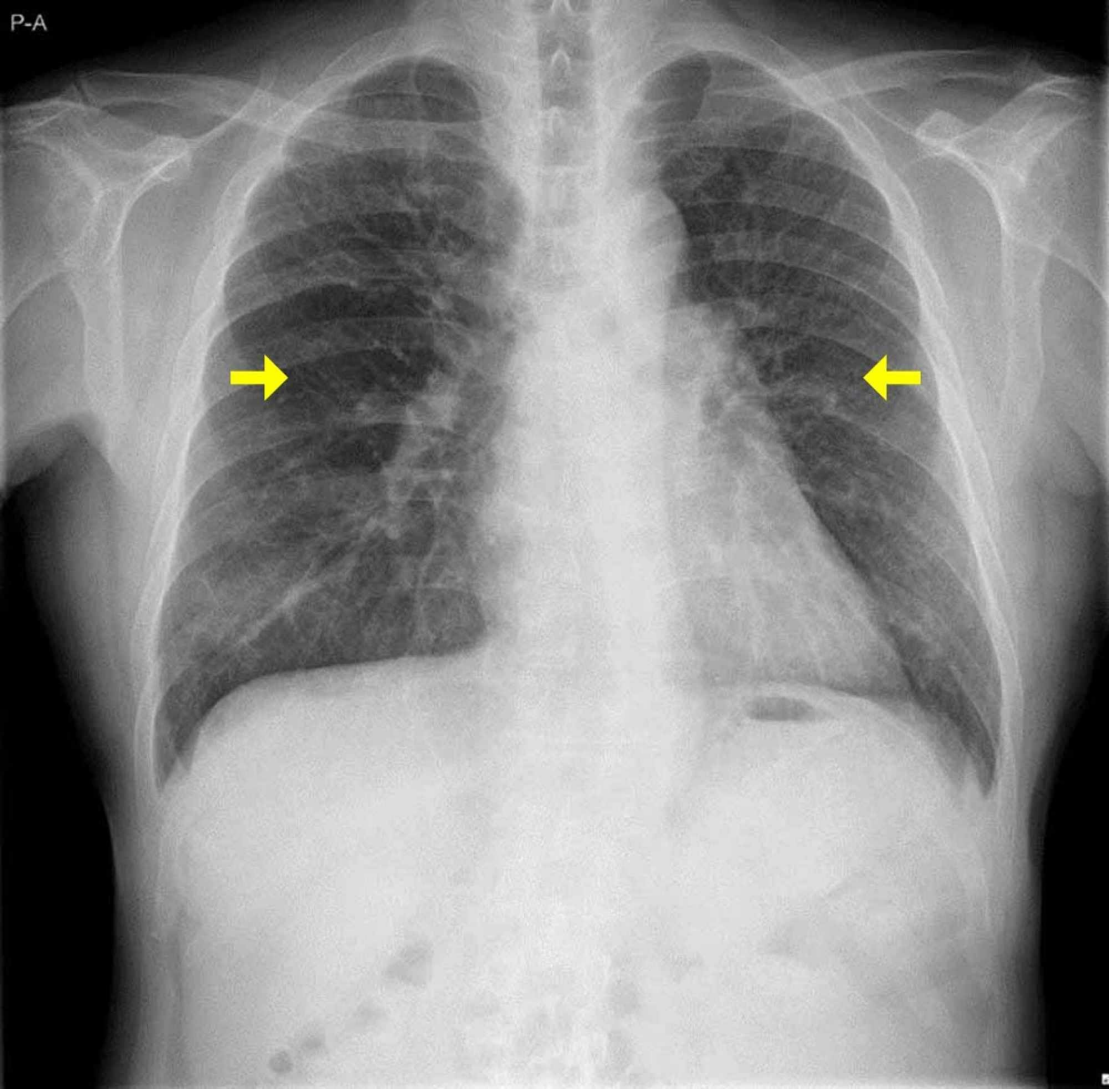
Chest X-ray before discharge from the hospital Chest X-ray showing normal lung field before discharge from the hospital.

On day 8, he was transferred to a ward with a Barthel index (BI) of 95 (stairs: need help). The six-minute walk test was 260 m (45% of normal value) on day 10 and 395 m (69%) on day 24 [10]. On day 22, chest X-ray showed normal lung fields (Figure 3).

After steroid tapering in a ward, he was discharged directly home with a BI of 100 on day 27.

## Discussion

We successfully treated this patient with awake ECMO at the early phase of ARDS. Although the patient experienced ongoing catabolism and muscle atrophy, we minimized the atrophy and weakness by mobilizing the patient during the use of ECMO. While the treatment of ARDS often requires deep sedation, ECMO can keep patients awake and make mobilization possible with multidisciplinary team involvement.

In this patient, the extent of atrophy was lower than in a previous report (13.0% vs. 20.7% on day 7), although the patient’s severity was the same (APACHE II [Acute Physiology And Chronic Health Evaluation II] score of 27 vs. 27.5 [23.0-29.3]) [11]. Moreover, this patient was treated with steroids, which is a risk factor for muscle atrophy [6]. Rehabilitation is important to prevent muscle atrophy [6]. In this case, the patient was safely mobilized during ECMO treatment. However, in some facilities, patients with ECMO are not always mobilized due to a fear of cannula dislodgement [12]. In a previous study, patients with ECMO were safely mobilized out of bed with standing and ambulation even under femoral cannulation [13]. ECMO is not a barrier to mobilization and can be used to attenuate muscle atrophy with mobilization.

Diaphragm dysfunction is a critical problem in mechanically ventilated patients. Diaphragm atrophy occurs in around 60% of mechanically ventilated patients [8]. Prevention of diaphragm atrophy is gaining increased attention with diaphragm-protective ventilation, but no study clearly reported the prevention of diaphragm atrophy [6]. Avoiding high inspiratory pressure is promising in an observational study [14]. In clinical practice, it is difficult to suppress inspiratory pressure due to the complication of hypercapnia. ECMO can make limited inspiratory pressure possible to protect both the lungs and diaphragm [7]. Moreover, a recent study reported that ultra-protective lung ventilation (target tidal volume: 4 mL/kg IBW) was more effective than conventional lung-protective ventilation for lung injury [15]. In our case report, the tidal volume was managed to 4.0-4.6 mL/kg, which is close to ultra-protective ventilation [16]. This ultra-protective ventilation with the use of ECMO might be protective to diaphragm as well as lungs because this patient did not show the change of diaphragm muscle thickness. Of course, further clinical trials will be needed to confirm it.

In our patient, BUN/Cr and urinary titin were higher than the normal level. BUN/Cr is a marker of catabolism in critically ill patients [17]. Similarly, titin is a spring-like protein in muscle and can be used as a marker of muscle catabolism [18]. Although this patient had elevated catabolism, the muscle atrophy was lower than in a previous study. Moreover, physical function was maintained during the ICU stay. At discharge from the ICU, the MRC score had a full score of 60, and a score greater than 55 has been associated with a better five-year mortality ratio [19]. Finally, at hospital discharge, the patient did not have deteriorated activity of daily living with a BI of 100, and the six-minute walk test was better than previous reports [20]. Awake ECMO and early mobilization possibly contributed to this physical function.

Of course, the early initiation of ECMO is still controversial. In this patient, neuromuscular-blocking agents or prone positioning may have been chosen in a different facility, but these treatments require the deep sedation of patients with unknown long-term outcomes. In this case report, the patient returned directly home without prominent physical dysfunction. We need to choose proper treatment in each patient to prevent physical dysfunction as well as lung injury. Awake ECMO is promising for better outcomes, and we need further research to investigate if ECMO can improve long-term outcomes.

## Conclusions

ARDS causes prominent muscle atrophy and weakness because conventional treatment of ARDS often requires deep sedation and immobilization. ECMO can keep patients awake and make mobilization possible. The findings in our case suggest that the early initiation of ECMO can accompany mobilization and attenuate muscle atrophy and weakness in ARDS.
